# Non-linear relationship between high-density lipoprotein cholesterol and metabolic dysfunction-associated steatotic liver disease: a cross-sectional study in the Japanese population

**DOI:** 10.3389/fmed.2026.1751477

**Published:** 2026-02-10

**Authors:** Xiang Guo, Yucong Zou, Cui Yang, Changchun Cao, Yulong Wang, Fubing Zha

**Affiliations:** 1 Department of Rehabilitation, Shenzhen Second People's Hospital, The First Affiliated Hospital of Shenzhen University, Shenzhen, Guangdong, China; 2 Department of Rehabilitation, Shenzhen Dapeng New District Nan'ao People's Hospital, Shenzhen, Guangdong, China

**Keywords:** generalized additive model, high-density lipoprotein cholesterol, lipid, metabolic dysfunction-associated steatotic liver disease, non-linearity

## Abstract

**Background:**

Research into the effect of high-density lipoprotein cholesterol (HDL-C) on metabolic dysfunction-associated steatotic liver disease (MASLD) remains relatively limited. This research aims to shed light on how HDL-C levels correlate with MASLD among the Japanese demographic.

**Methods:**

A comprehensive review of health records from 14,280 patients at Murakami Memorial Hospital from 2004 to 2015 was undertaken. To investigate the linear association of HDL-C concentrations with MASLD occurrence, binary logistic regression was applied. Additionally, a generalized additive model (GAM) integrated with smooth curve fitting procedures was implemented to characterize potential non-linear dependencies.

**Results:**

An inverse correlation was observed between HDL-C levels and MASLD prevalence (OR = 0.46; 95% CI: 0.37–0.58), holding steady after adjustments for various demographic and health variables. The consistency of these findings was confirmed through multiple sensitivity tests. The study also uncovered a non-linear correlation between HDL-C concentrations and MASLD occurrence. A detailed analysis using a two-piece logistic regression and recursive techniques pinpointed a critical HDL-C level of 1.04 mmol/L. Above this level, each unit increase in HDL-C was linked to a 61% decrease in the likelihood of MASLD (OR = 0.39; 95% CI: 0.30–0.50), a connection that dissipates below this threshold of HDL-C (OR = 1.49; 95% CI: 0.66–3.35).

**Conclusion:**

The investigation revealed an inverse, non-linear relationship between HDL-C and MASLD within the Japanese community, emphasizing a pivotal threshold effect. Elevated HDL-C levels beyond 1.04 mmol/L significantly diminish MASLD risk.

## Introduction

MASLD has emerged as the predominant chronic liver condition globally in recent years. Characterized by the accumulation of lipids within the liver and subsequent inflammatory responses, MASLD precipitates structural and functional impairments within the hepatic architecture (1–3). Beyond its immediate impact on liver health, MASLD exerts deleterious effects on various other organs via both intra- and extrahepatic pathways of metabolic dysregulation (4, 5). The rising global burden of MASLD, which currently afflicts approximately one-fourth of the adult population worldwide (1, 6), underscores its significance as a pressing public health issue. Consequently, delineating the trajectory of MASLD within the population emerges as a critical endeavor.

Much previous evidence demonstrated that abnormalities in lipid metabolism, including elevated levels of triglycerides (TG) and decreased levels of HDL-C, were strongly associated with MASLD (7). HDL-C, transported by high-density lipoprotein particles, is crucial for the transportation and metabolism of cholesterol, aiding in the removal of surplus cholesterol from the body’s peripheral tissues to the liver for further processing. Numerous studies highlight the protective qualities of HDL-C against atherosclerosis and inflammation, demonstrating its negative relationship with the incidence of cardiovascular diseases and confirming its essential role in safeguarding against cardiovascular disorders (8, 9). Current evidence substantiates a correlation between increased HDL-C levels and decreased MASLD risk (10, 11). However, some reports in the literature present conflicting results regarding this association (12–14). Recent investigations, including two retrospective cohort studies, have reported no discernible differences in HDL-C concentrations between patients with MASLD and those with non-MASLD (12, 13). Furthermore, a prospective cohort study conducted in Israel with 349 participants demonstrated that higher concentrations of HDL-C were not associated with a statistically significant reduction in incident MASLD risk (14). In light of these conflicting observations, we utilize a Japanese public database to elucidate the effect of HDL-C on MASLD within a cohort.

## Methods

### Study source

Scholars have the opportunity to freely reference and utilize primary data from the Dryad Digital Repository. For our analysis, we engaged with the initial data made available by Takuro Okamura et al. (15), sourced directly from the Dryad data archive. This dataset could be subjected to a subsequent analytical process in compliance with the stipulations set forth by Dryad’s usage policy. Our study constituted a secondary analysis, leveraging publicly accessible data from a medical examination program.

### Study participants

Each subject provided written informed consent in the original investigation, which received the green light from the Clinical Research Ethics Committee of Murakami Memorial Hospital (15). Additionally, this research was conducted in strict compliance with the ethical guidelines established by the Declaration of Helsinki. All methodological procedures detailed in the Declarations section adhered to applicable regulatory standards and international guidelines. In addition, the study has also been approved by the Ethics Committee of the Shenzhen Dapeng New District Nan’ao People’s Hospital (2022082201).

**Figure 1:**
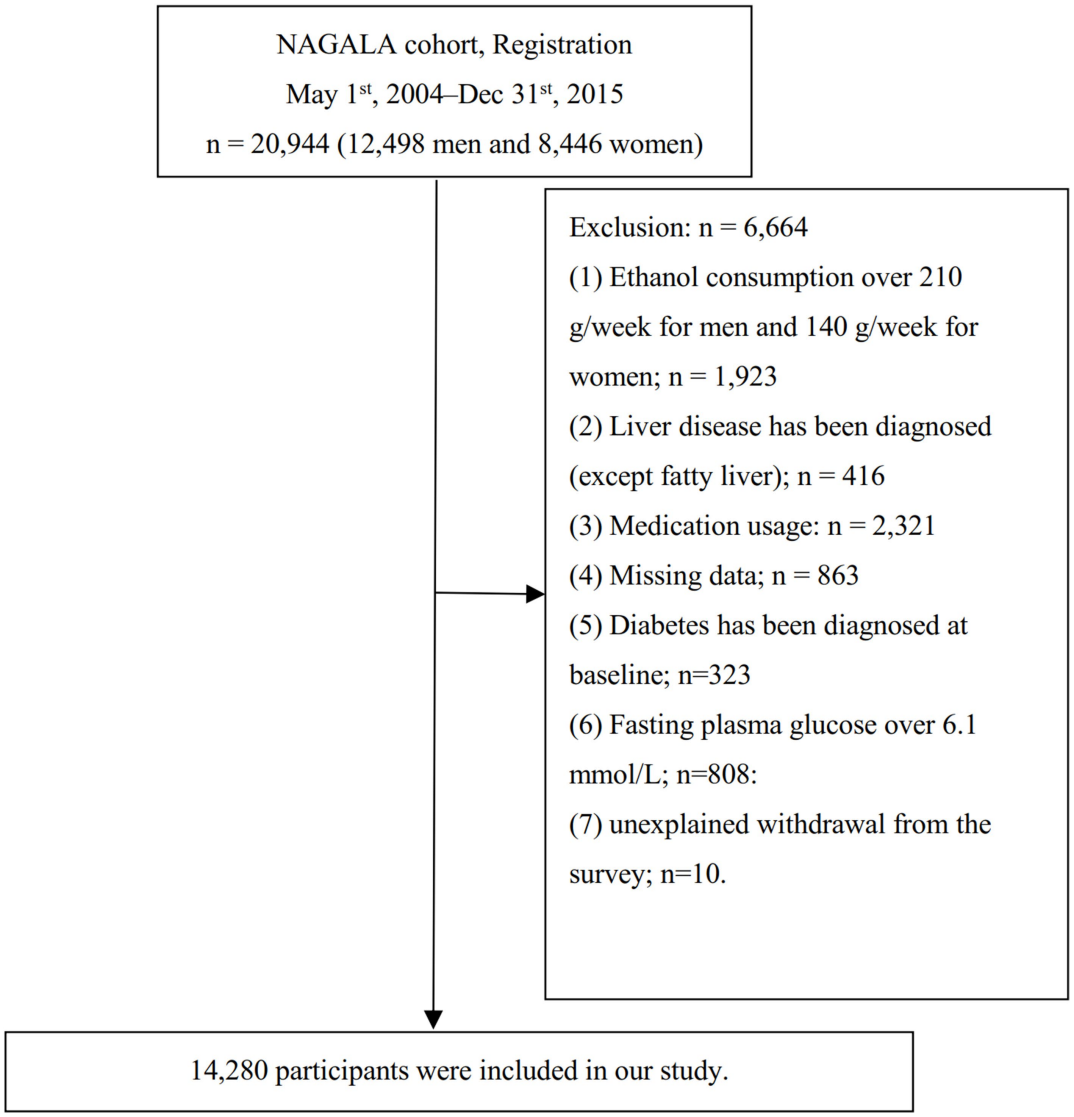
Study population.

From 2004 to 2015, 20,944 individuals undergoing routine health examinations and completing two or more assessments were enrolled. Following the exclusion of 6,664 individuals for various reasons, the analysis proceeded with 14,280 subjects, as detailed in Figure 1. Exclusion criteria encompassed the following predefined parameters, including (1) those with ethanol consumption exceeding 30 g/d for male or 20 g/d for female (*n* = 1,923); (2) diagnosed with liver diseases other than fatty liver (*n* = 416); (3) incomplete dataset for required data (*n* = 863); (4) current medication use (*n* = 2,321); (5) diagnosed DM or FPG levels equal to or exceeding 6.1 mmol/L (*n* = 1,131); (6) exited the study for reasons not disclosed (*n* = 10).

### Covariates

The covariate selection was informed by clinical expertise and extant empirical evidence, ensuring methodologically robust variable specification. Considered covariates included: (1) categorical variables encompassed sex, physical activity and smoking status; (2) continuous variables such as ethanol consumption, systolic blood pressure (SBP), alanine aminotransferase (ALT), TC, aspartate aminotransferase (AST), fasting plasma glucose (FPG), TG, glycosylated hemoglobin A1c (HbA1c), age, diastolic blood pressure (DBP), and body mass index (BMI). To capture participant data, two primary methods were employed: a structured self-administered questionnaire for lifestyle and medical history, and a precise physical assessment conducted by trained personnel for anthropometric measures (weight, height, and blood pressure). Furthermore, the original study’s team uniformly collected laboratory data, including HDL-C, TC, FPG, HbA1c, AST, ALT, and TG, under strict quality control conditions.

### Diagnosis of incident MASLD

The dependent variable under scrutiny in our research was MASLD. Diagnostic procedures for MASLD entailed conducting liver ultrasonography while meticulously excluding subjects with a history of significant ethanol consumption or previously identified etiologies of liver pathology (16). Ultrasonographic assessments were performed by qualified technicians, while definitive diagnoses were established by gastroenterology specialists. The diagnostic evaluation involved a comprehensive assessment of key sonographic features, such as hepatic echogenicity, hepatorenal echogenic contrast, vascular clarity, and degree of ultrasonic attenuation. This analytical process was carried out independently, without reference to additional participant information (16).

### Statistical analysis

All study participants were categorized based on HDL-C quartiles for the analysis of baseline characteristics. Continuous variables were summarized as mean ± standard deviation for normally distributed data or median (interquartile range) for skewed data. Group comparisons across HDL-C quartiles were performed using the One-Way ANOVA for normally distributed continuous variables, the Kruskal–Wallis H test for non-normally distributed continuous variables, and the Chi-square test (χ^2^) for categorical variables.

Our investigation delved into the effect of HDL-C on MASLD through logistic regression model analyses. This analytical approach was structured around three models: Model 1, which did not adjust for any variables; Model 2, which adjusted for smoking status, ethanol consumption, DBP, age, BMI, SBP, and physical activity; and Model 3, which further adjusted for ALT, TC, FPG, AST, HbA1c, and TG, in addition to the variables accounted for in Model 2. Throughout this analysis, we calculated and reported the odds ratios and 95% confidence intervals. Adjustments were specifically made for those covariates that, upon inclusion in the model, resulted in a change of ≥10% in the matching hazard ratio (17).

Upon recognizing that binary logistic regression might inadequately capture complex non-linear associations, this study applied generalized additive models (GAM) with smooth curve fitting to explore the relationship between HDL-C levels and MASLD risk. When non-linearity was detected, a recursive algorithm was used to identify the inflection point. Subsequently, a two-piece logistic regression model was constructed on both sides of this threshold. Model selection was guided by the log-likelihood ratio test, with the optimal model chosen based on the smallest *p*-value.

An extensive array of sensitivity analyses was undertaken to substantiate the robustness of our outcomes. Acknowledging the constraints of generalized linear models in representing non-linear relationships, a GAM was implemented within Model 4 to incorporate adjustments for covariates. Additionally, sensitivity analyses were broadened to exclude individuals with a BMI ≥ 25 kg/m^2^ or aged ≥60 years, further exploring the linkage between HDL-C levels and MASLD risk. E-values were computed in anticipation of potential unmeasured confounding factors that could influence the HDL-C-MASLD risk nexus (18).

These analytical procedures were conducted utilizing the R statistical software and the Empower Stats. Statistical significance was adjudicated based on a *p*-value criterion of less than 0.05 (two-tailed).

## Results

Table 1 provides a detailed overview of clinical, biochemical, and additional parameters measured across all participants. The cohort for the final analysis comprised 14,280 subjects, with a mean age of 43.53 ± 8.89 years and a male representation of 52.10%. Within this cohort, MASLD was diagnosed in 2,515 individuals. The average values for BMI and HDL-C were reported as 22.07 ± 3.14 kg/m^2^ and 1.46 ± 0.40 mmol/L, respectively. The study further stratified participants into quartiles based on their HDL-C levels for comparative analysis. Significantly, the analysis revealed that participants within the highest quartile of HDL-C levels (Q4 group) demonstrated markedly lower measures of DBP, HbA1c, ethanol consumption, age, SBP, ALT, FPG, TG, BMI, AST, and TC. This group (Q4 group) also featured a reduced proportion of male participants and individuals who smoke. Additionally, it was observed that these individuals exhibited the highest prevalence of physical activity.

**Table 1:** The baseline characteristics of participants.

HDL-C (mmol/L)	Q1 (≤1.16)	Q2 (1.16 to ≤1.41)	Q3 (1.41 to ≤1.70)	Q4 (>1.70)	*p*-value
Participants	3,491	3,649	3,569	3,571	
Gender					<0.001
Women	566 (16.21%)	1,426 (39.08%)	2,105 (58.98%)	2,743 (76.81%)	
Men	2,925 (83.79%)	2,223 (60.92%)	1,464 (41.02%)	828 (23.19%)	
Age (years)	44.33 ± 9.03	43.45 ± 8.94	43.08 ± 8.74	43.29 ± 8.81	<0.001
Ethanol consumption (g/week)	1 (0–54)	1 (0–54)	1 (0–36)	1 (0–23.69)	<0.001
Smoking status					<0.001
Never-smoker	1,402 (40.16%)	2080 (57.00%)	2,451 (68.67%)	2,818 (78.91%)	
Ex-smoker	781 (22.37%)	742 (20.33%)	586 (16.42%)	463 (12.97%)	
Current-smoker	1,308 (37.47%)	827 (22.66%)	532 (14.91%)	290 (8.12%)	
Physical activity					<0.001
No	2,964 (84.90%)	3,025 (82.90%)	2,946 (82.54%)	2,869 (80.34%)	
Yes	527 (15.10%)	624 (17.10%)	623 (17.46%)	702 (19.66%)	
SBP (mmHg)	118.39 ± 14.58	115.41 ± 14.96	112.01 ± 14.26	110.10 ± 14.15	<0.001
DBP (mmHg)	74.60 ± 10.10	72.19 ± 10.34	69.74 ± 10.01	68.08 ± 9.93	<0.001
BMI (kg/m^2^)	23.93 ± 3.14	22.54 ± 3.09	21.40 ± 2.72	20.44 ± 2.42	<0.001
ALT (IU/L)	21 (16–30)	17 (13–24)	15 (12–20)	14 (11–18)	<0.001
AST (IU/L)	18 (15–22)	17 (14–21)	17 (14–20)	16 (13–20)	<0.001
HDL-C (mmol/L)	0.99 ± 0.13	1.29 ± 0.07	1.55 ± 0.08	2.00 ± 0.27	<0.001
TG (mmol/L)	1.19 (0.81–1.69)	0.80 (0.56–1.14)	0.63 (0.44–0.87)	0.52 (0.38–0.70)	<0.001
TC (mmol/L)	5.06 ± 0.90	5.06 ± 0.90	5.08 ± 0.84	5.30 ± 0.80	<0.001
HbA1c (%)	5.19 ± 0.34	5.18 ± 0.32	5.15 ± 0.31	5.18 ± 0.30	<0.001
FPG (mmol/L)	5.29 ± 0.38	5.18 ± 0.40	5.09 ± 0.41	5.03 ± 0.41	<0.001

Values are *n* (%), mean ± SD, or median (quartile).

SBP, systolic blood pressures; DBP, diastolic blood pressures; BMI, body mass index; ALT, alanine aminotransferase; AST, aspartate aminotransferase; HDL-C, high-density lipoprotein cholesterol; TC, total cholesterol; TG, triglycerides; HbA1c, hemoglobin A1c; FPG, fasting plasma glucose.

### The effect of HDL-C on MASLD

Table 2 meticulously presents the findings from a binary logistic regression analysis, elucidating the OR and 95% CI pertinent to the effect of HDL-C on MASLD. In Model 1, which refrained from adjusting for extraneous variables, a significant inverse association was observed between HDL-C levels and MASLD risk (OR: 0.05, 95% CI: 0.05–0.06). Upon the inclusion of adjustments for smoking status, DBP, age, ethanol consumption, SBP, BMI, and physical activity in Model 2, this correlation still persisted (OR: 0.28, 95% CI: 0.23–0.33). After further adjusting for HbA1c, TC, ALT, FPG, AST, and TG, in addition to the variables accounted for in Model 2, the inverse relationship between HDL-C levels and MASLD risk could also be revealed (OR: 0.46, 95% CI: 0.37–0.58) in Model 3.

**Table 2:** Relationship between HDL-C and MASLD risk in different models.

Variable	Model 1 (OR, 95% CI, *p*)	Model 2 (OR, 95% CI, *p*)	Model 3 (OR, 95% CI, *p*)	Model 4 (OR, 95% CI, *p*)
HDL-C	0.05 (0.05, 0.06) < 0.0001	0.28 (0.23, 0.33) < 0.0001	0.46 (0.37, 0.58) < 0.0001	0.59 (0.46, 0.74) < 0.0001
HDL-C (quartile)				
Q1	ref	ref	ref	ref
Q2	0.43 (0.39, 0.48) < 0.0001	0.71 (0.63, 0.81) < 0.0001	0.91 (0.78, 1.04) 0.1728	0.97 (0.83, 1.12) 0.6410
Q3	0.16 (0.14, 0.19) < 0.0001	0.44 (0.38, 0.52) < 0.0001	0.63 (0.53, 0.76) < 0.0001	0.73 (0.60, 0.88) 0.0009
Q4	0.07 (0.05, 0.08) < 0.0001	0.31 (0.25, 0.39) < 0.0001	0.46 (0.36, 0.59) < 0.0001	0.58 (0.45, 0.75) < 0.0001
*p* for trend	<0.0001	<0.0001	<0.0001	<0.0001

Model 1: we did not adjust for other covariants.

Model 2: we adjusted for age, ethanol consumption, smoking, physical activity SBP, DBP, and BMI.

Model 3: we adjusted for age, ethanol consumption, smoking, physical activity, SBP, DBP, BMI, TC, TG, HbA1c, and FPG.

Model 4: we adjusted for age, ethanol consumption, smoking, physical activity, SBP, DBP, BMI, TC, TG, HbA1c, and FPG. However, continuous covariates were adjusted as nonlinearity.

OR, Odds ratios; CI, Confidence interval: Ref, Reference.

Moreover, a detailed examination stratified by quartiles of HDL-C levels, with the lowest quartile (Q1) designated as the reference category, revealed a progressive decrement in the OR associated with ascending quartiles. This gradient effect was elucidated by the observed OR across the quartiles: for the second quartile (Q2), an OR of 0.91 (95% CI: 0.78–1.04) was reported; for the third quartile (Q3), the OR was 0.63 (95% CI: 0.53–0.76); and for the fourth quartile (Q4), the OR further decreased to 0.46 (95% CI: 0.36–0.59), as detailed in Table 2, Model 3.

### Sensitive analysis

To incorporate the continuous covariate (HDL-C) into the equation, we applied a Generalized Additive Model (GAM) using a smooth function. The results from the fully adjusted Model 4, as presented in Table 2 (OR: 0.59, 95% CI: 0.46–0.74), demonstrated a consistent relationship. To assess the robustness of this association to unmeasured confounding, we calculated an E-value. The obtained E-value of 2.31 exceeds the relative risk that unmeasured confounders would need to have with both the exposure and outcome, suggesting that such confounding is unlikely to fully explain the observed association between HDL-C and MASLD.

To evaluate the robustness of our findings, we performed sensitivity analyses. After excluding participants with a BMI ≥ 25 kg/m^2^, the inverse association between HDL-C and MASLD persisted following multivariable adjustment (OR = 0.46, 95% CI: 0.35–0.61; Model 5, Table 3). Similarly, after excluding those aged 60 years or older, the results remained consistent (OR = 0.44, 95% CI: 0.35–0.55; Model 6, Table 3). The consistency across these analyses strengthens the reliability of our primary finding.

**Table 3:** Relationship between HDL-C and MASLD risk in different sensitivity analyses.

Exposure	Model 5 (OR, 95% CI, *p*)	Model 6 (OR, 95% CI, *p*)
HDL-C	0.46 (0.35, 0.61) < 0.0001	0.44 (0.35, 0.55) < 0.0001
HDL-C (quartile)		
Q1	ref	ref
Q2	0.92 (0.77, 1.10) 0.3828	0.90 (0.77, 1.04) 0.1500
Q3	0.61 (0.49, 0.76) < 0.0001	0.62 (0.52, 0.75) < 0.0001
Q4	0.48 (0.36, 0.64) < 0.0001	0.43 (0.34, 0.56) < 0.0001
*p* for trend	<0.0001	<0.0001

Model 5 was sensitivity analysis after excluding those with BMI ≥ 25 kg/m^2^. We adjusted age, ethanol consumption, smoking, physical activity, SBP, DBP, BMI, TC, TG, HbA1c, and FPG.

Model 6 was sensitivity analysis after excluding those with age ≥ 60 years. We adjusted age, ethanol consumption, smoking, physical activity, SBP, DBP, BMI, TC, TG, HbA1c, and FPG.

OR, Odds ratios; CI, Confidence interval; Ref, Reference.

### The analyses of the non-linear relationship

**Figure 2:**
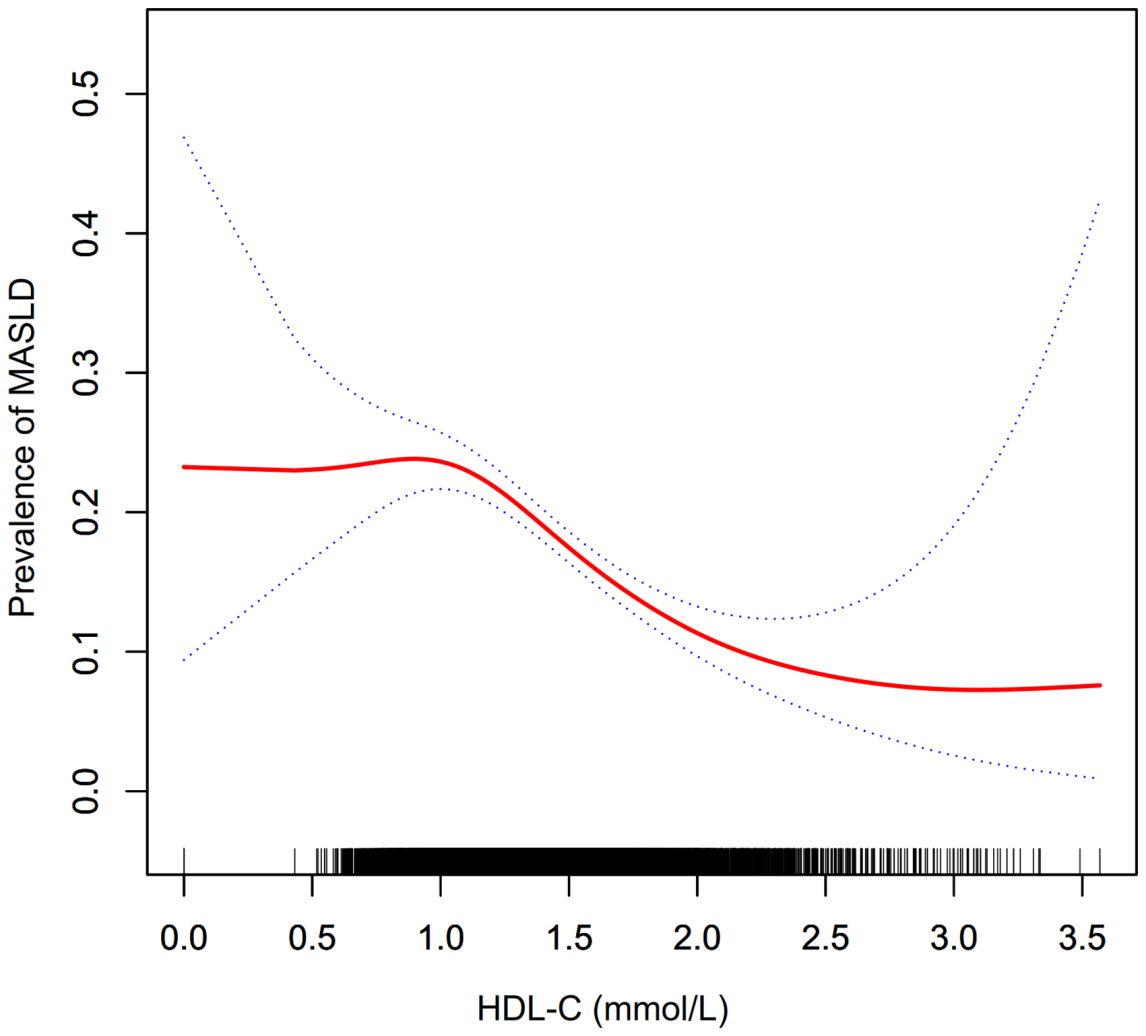
The nonlinear relationship between the HDL-C and prevalence of MASLD. A nonlinear relationship was detected after adjusting for age, ethanol consumption, smoking, physical activity, SBP, DBP, BMI, TC, TG, HbA1c, and FPG.

Figure 2 elucidated the nonlinear association between HDL-C levels and the risk of MASLD. A nonlinear correlation between HDL-C levels and MASLD risk was observed upon adjustment for confounding covariates, as detailed in Table 4. The inflection point for HDL-C was identified at 1.04 mmol/L utilizing a two-piecewise logistic regression model, with the log-likelihood ratio test yielding a significance level of (*p* = 0.003). For HDL-C levels exceeding 1.04 mmol/L, an inverse relationship with the incidence of MASLD was noted (OR: 0.39, 95% CI: 0.30–0.50). Conversely, at HDL-C levels of 1.04 mmol/L or below, the association with MASLD incidence did not reach statistical significance (OR: 1.49, 95% CI: 0.66–3.35).

**Table 4:** The result of the two-piecewise logistic regression model for MASLD.

Incident MASLD	OR (95% CI)	*p*-value
Fitting model by standard logistic regression	0.46 (0.37, 0.58)	<0.0001
Fitting model by two-piecewise logistic regression		
The inflection point of HDL-C	1.04 mmol/L	
≤1.04 mmol/L	1.49 (0.66, 3.35)	0.3332
>1.04 mmol/L	0.39 (0.30, 0.50)	<0.0001
*p* for log-likelihood ratio test	0.003	

This model was adjusted for age, ethanol consumption, smoking, physical activity, SBP, DBP, BMI, TC, TG, HbA1c, and FPG.

OR, Odds ratios; CI, Confidence interval; Ref, Reference.

## Discussion

In this cross-sectional study, we identified a significant inverse correlation between elevated HDL-C levels and MASLD risk after adjusting for potential confounders. This relationship was characterized by a non-linear pattern, with an inflection point at 1.04 mmol/L. Above this threshold, HDL-C demonstrated a statistically significant negative association with MASLD incidence. In contrast, the association was not significant below this level.

Numerous studies have shown that lipid metabolism disorders are closely related to the development and progression of MASLD (19–21). HDL-C is an important indicator for assessing lipid metabolism disorders and one of the diagnostic criteria for metabolic syndrome (22). Despite established evidence linking a decreased HDL-C to heightened diabetes mellitus and metabolic syndrome risk, its association with MASLD risk remains a subject of ongoing debate. A prospective cohort survey that included 32,121 participants, adjusted for sex, age, profession, and education, found that HDL-C was negatively associated with MASLD (10). In addition, another prospective cohort study involving 11,891 non-obese participants also identified HDL-C as a protective factor for MASLD (11). However, discordant findings exist within the literature. A retrospective cohort study encompassing a total of 60 MASLD patients was executed at the Abant Izzet Baysal University Hospital, specifically within the Department of Internal Medicine and Gastroenterology (12). Concurrently, the study incorporated 57 healthy individuals as a control cohort to facilitate a comparative analysis (12). Through meticulous statistical examination, the assessment of HDL-C levels between individuals diagnosed with MASLD and those with non-MASLD demonstrated no statistically significant disparity (12). Another study involving 357 children and adolescents aged 2–18 years with obesity found no difference in HDL-C levels between patients with and without MASLD (13). Furthermore, a prospective cohort investigation conducted in Israel, encompassing a total of 349 participants, elucidated that the elevation in HDL-C levels does not statistically correlate with a decreased risk of developing MASLD (14). The present investigation fortifies the discourse within existing scholarly literature, positing a negative association between elevated levels of HDL-C and the risk of MASLD. The observed discrepancies in findings across various studies may be attributed to several factors, including variations in gender distribution, demographic characteristics of study cohorts, the range of HDL-C levels examined, and the covariates adjusted for in the analyses. Furthermore, it is imperative to acknowledge that the outcomes derived from linear regression analyses may be susceptible to distortion by nonlinear relationships, thereby affecting the integrity of the established linear associations. Consequently, the heterogeneous results reported in the literature may partly stem from a nonlinear relationship between HDL-C levels and the incidence of MASLD.

The exact biological pathways through which HDL-C levels influence the development and progression of MASLD remain incompletely elucidated. However, several potential mechanisms have been proposed to account for the contributory role of HDL-C in MASLD pathogenesis. HDL-C possesses anti-inflammatory properties that mitigate hepatic inflammation, a key driver of MASLD progression to more severe forms like non-alcoholic steatohepatitis (23). HDL-C is known for its antioxidant capabilities, reducing oxidative stress within hepatocytes (24). Oxidative stress is a critical factor in the pathogenesis of MASLD, leading to lipid peroxidation and further liver injury (24). By improving insulin sensitivity, HDL-C helps reduce hepatic fat accumulation (25). HDL-C plays a significant role in regulating lipid metabolism, including promoting cholesterol efflux from hepatocytes, thus reducing the lipid content within the liver and preventing the progression of MASLD (26).

Moreover, our analysis elucidated a nonlinear correlation between HDL-C levels and MASLD risk. Upon adjusting for a spectrum of confounders, the inflection point for HDL-C levels was pinpointed at 1.04 mmol/L. Notably, at HDL-C concentrations exceeding this threshold, each unit increment in HDL-C levels was correlated with a 61% reduction in the adjusted OR for MASLD risk (OR = 0.39, 95% CI: 0.30–0.50). Conversely, at HDL-C levels of 1.04 mmol/L or lower, no significant association was discerned between elevated HDL-C levels and the risk of MASLD (OR = 1.49, 95% CI: 0.66–3.35). The observed nonlinear relevance between HDL-C levels and MASLD risk may be attributable to the modulating effects of other covariates at baseline. It was observed that participants situated to the left of the inflection point typically exhibited older age demographics and elevated levels of TC, LDL-C, ALT, AST, HbA1c, and FPG, alongside a higher prevalence of current ethanol consumption (Supplementary Table S1). Prior research has implicated that these covariates were risk factors for the development of MASLD (27–29). Consequently, we postulated that the protective role of HDL-C against MASLD may be diminished below the inflection point due to the effects of increased risk factors for MASLD, such as age, TC, LDL-C, ALT, AST, HbA1c, FPG, and ethanol consumption. Identifying a curved relationship between HDL-C and MASLD risk can help with clinical recommendations and decision-making to improve preventive strategies for MASLD.

This investigation presents numerous advantages. (1) It employs a GAM alongside smooth curve fitting techniques to delve into the nonlinear relationship under study, offering insights of significant clinical relevance previously unaddressed in the literature. (2) The application of rigorous statistical methods has effectively reduced the impact of residual confounding variables. (3) The integrity of our findings is further substantiated by a comprehensive suite of sensitivity analyses, including the transformation of the independent variable of interest, the incorporation of continuity covariates into the model as curves through the use of a GAM, and the computation of E-values to assess the influence of potential unmeasured confounding factors.

Nevertheless, the study is not without its limitations, which warrant careful consideration. Due to its cross-sectional design, it is challenging to establish a direct causal link between variables. Moreover, the generalizability of our conclusions may be limited, as the cohort consists solely of Japanese participants, thus potentially excluding applicability to other racial groups, geographical regions, and specific demographic segments. Future research endeavors might benefit from a broader scope, including diverse populations and ethnic backgrounds, possibly through new study designs or collaborations to investigate the HDL-C and MASLD risk correlation across different demographics. Furthermore, despite efforts to control for known confounders such as FPG, HbA1c, and BMI, the inherent nature of observational studies means that the presence of uncontrolled or unidentified confounding factors cannot be entirely ruled out. Nonetheless, the calculated E-values suggest that such unmeasured confounders are unlikely to alter our findings significantly.

## Conclusion

This investigation elucidates a non-linear and inversely proportional relationship between HDL-C and MASLD risk. Notably, a significant inverse correlation with MASLD risk emerges when HDL-C levels surpass the threshold of 1.04 mmol/L. These findings are anticipated to be a pivotal reference for clinical practitioners in managing HDL-C levels. From a therapeutic standpoint, augmenting HDL-C levels beyond this critical inflection point appears to be a rational strategy. Elevating HDL-C to levels above this threshold is associated with a marked diminution in the risk of developing MASLD.

## Data Availability

The datasets presented in this study can be found in online repositories. The names of the repository/repositories and accession number(s) can be found at: https://datadryad.org/stash/dataset/doi:10.5061/dryad.8q0p192.
