# Supplementary material for: Non-linear relationship between high-density lipoprotein cholesterol and metabolic dysfunction-associated steatotic liver disease: a cross-sectional study in the Japanese population
Source: Front Med (Lausanne). 2026 Feb 10;13:1751477. doi: 10.3389/fmed.2026.1751477 (PMC12929366; doi:10.3389/fmed.2026.1751477)
Supplement: Supplementary file 1 [file Table_1.docx]

**Table S1** The Baseline Characteristics of participants based on inflection point for HDL-C

| HDL-C (mmol/L) | ≤1.04 | >1.04 | P-value |
| --- | --- | --- | --- |
| Participants | 1977 | 12303 |  |
| Gender |  |  | <0.001 |
| Women | 242 (12.24%) | 6598 (53.63%) |  |
| Men | 1735 (87.76%) | 5705 (46.37%) |  |
| Age(years) | 44.44 ± 8.98 | 43.39 ± 8.87 | <0.001 |
| Ethanol consumption(g/week) | 1 (0-45.3) | 1 (0-36) | <0.001 |
| Smoking status |  |  | <0.001 |
| Never-smoker | 718 (36.32%) | 8033 (65.29%) |  |
| Ex-smoker | 434 (21.95%) | 2138 (17.38%) |  |
| Current-smoker | 825 (41.73%) | 2132 (17.33%) |  |
| Regular exerciser |  |  | <0.001 |
| No | 1696 (85.79%) | 10108 (82.16%) |  |
| Yes | 281 (14.21%) | 2195 (17.84%) |  |
| SBP (mmHg) | 119.14 ± 14.72 | 113.13 ± 14.68 | <0.001 |
| DBP (mmHg) | 75.06 ± 10.22 | 70.51 ± 10.28 | <0.001 |
| BMI (kg/m^2^) | 24.27 ± 3.06 | 21.71 ± 3.00 | <0.001 |
| ALT (IU/L) | 22 (16-31) | 16 (12-21) | <0.001 |
| AST (IU/L) | 19 (15-23) | 17.00 (14-21) | <0.001 |
| HDL-C (mmol/L) | 0.91 ± 0.12 | 1.55 ± 0.36 | <0.001 |
| TG (mmol/L) | 1.56 ± 0.95 | 0.78 ± 0.49 | <0.001 |
| TC (mmol/L) | 5.04 ± 0.91 | 5.14 ± 0.86 | <0.001 |
| HbA1c (%) | 5.19 ± 0.35 | 5.18 ± 0.32 | 0.046 |
| FPG (mmol/L) | 5.31 ± 0.37 | 5.12 ± 0.41 | <0.001 |

Values are n (%), mean ± SD, or median (quartile).

SBP: systolic blood pressures; DBP: diastolic blood pressures; BMI: body mass index; ALT: alanine aminotransferase; AST: aspartate aminotransferase; HDL-C: high-density lipoprotein cholesterol; TC: total cholesterol; TG: triglycerides; HbA1c: hemoglobin A1c; FPG: fasting plasma glucose
